# Resveratrol Treatment Normalizes the Endothelial Function and Blood Pressure in Ovariectomized Rats

**DOI:** 10.5935/abc.20170012

**Published:** 2017-02

**Authors:** Victor Fabricio, Jorge Camargo Oishi, Bruna Gabriele Biffe, Leandro Dias Gonçalves Ruffoni, Karina Ana da Silva, Keico Okino Nonaka, Gerson Jhonatan Rodrigues

**Affiliations:** Universidade Federal de São Carlos (UFSCar), São Carlos, SP - Brazil

**Keywords:** Blood Pressure, Rats, Wistar, Resveratrol, Phytoestrogens, Ovariectomy, Endothelium, Vascular

## Abstract

**Background:**

Despite knowing that resveratrol has effects on blood vessels, blood pressure and that phytostrogens can also improve the endothelium-dependent relaxation/vasodilation, there are no reports of reveratrol's direct effect on the endothelial function and blood pressure of animals with estrogen deficit (mimicking post-menopausal increased blood pressure).

**Objective:**

To verify the effect of two different periods of preventive treatment with resveratrol on blood pressure and endothelial function in ovariectomized young adult rats.

**Methods:**

3-month old female Wistar rats were used and distributed in 6 groups: intact groups with 60 or 90 days, ovariectomized groups with 60 or 90 days, and ovariectomized treated with resveratrol (10 mg/kg of body weight per day) for 60 or 90 days. The number of days in each group corresponds to the duration of the experimental period. Vascular reactivity study was performed in abdominal aortic rings, systolic blood pressure was measured and serum nitric oxide (NO) concentration was quantified.

**Results:**

Ovariectomy induced blood pressure increase 60 and 90 days after surgery, whereas the endothelial function decreased only 90 days after surgery, with no difference in NO concentration among the groups. Only longer treatment (90 days) with resveratrol was able to improve the endothelial function and normalize blood pressure.

**Conclusion:**

Our results suggest that 90 days of treatment with resveratrol is able to improve the endothelial function and decrease blood pressure in ovariectomized rats.

## Introduction

The endothelium is a monolayer of tissue located inside the blood vessels and can have endocrine and paracrine functions, regulating vascular function by releasing trophic and vasoactive factors that regulate the vascular tone and even control the vascular wall inflammation.^1^ Endothelial dysfunction is characterized mainly by a direct or indirect decrease of nitric oxide (NO) bioavailability^2^.

NO release by the endothelium is modulated by several factors, including estrogen. This hormone is able to increase NO bioavailability and production through genomic and non-genomic factors. Among them, we can mention its action on estrogen receptor α (ERα) and the reduction of oxidative stress.^3,4^ Thus, the reduction of this hormone that is observed after menopause can lead to endothelial dysfunction with a consequent increase in blood pressure.

In order to reduce some negative effects of estrogen deficiency, hormone replacement therapy (HRT) is commonly indicated. However, studies indicate that this treatment may be associated with adverse cardiovascular events, increased risk of the development of breast cancer and deep vein thrombosis in women with a predisposition to these conditions.^3,5,6^

In an attempt to find alternatives to HRT with fewer side effects, resveratrol (3,4,5'-trihydroxystilbene) has shown promising effect because of its similarity to diethylstilbestrol (a synthetic estrogen) and can be regarded as a phytoestrogen. In addition, resveratrol can exert its action on estrogen receptors and may then be regarded as a SERM (selective estrogen receptor modulator).^7,9^

Despite knowing that both phytoestrogens and SERMs are reported in the literature as acute improvers of endothelium-dependent relaxation/vasodilation^4^ and that studies indicate the effect of resveratrol on blood pressure and blood vessels,^10,11^ there are not many reports of its direct effect on both the endothelial function and blood pressure in animals with estrogen deficit only. Thus, the objective of this study was to verify the effect of two different preventive treatment protocols with resveratrol on blood pressure and endothelial function in young ovariectomized female rats.

## Methods

### Animals and treatments

The experimental protocol was performed in accordance with the guidelines of the Brazilian College for Animal Experimentation (COBEA) and was approved by the Ethics Committee of the Federal University of Sao Carlos - UFSCar (2-043/2013).

Sixty Wistar (*Rattus norvegicus albinis)* female rats (90 days old at the beginning of the experiment) were housed under controlled dark-light cycles (14h/10h from 6:00 pm to 8:00 am) and temperature (22 ± 2 °C) receiving standard diet and water *ad libitum* for 60 or 90 days.

The animals were randomly assigned to six experimental groups: intact - 60 Days Days (INT 60), ovariectomized - 60 days (OVX 60), ovariectomized + resveratrol - 60 days (OVX + RES 60), intact - 90 days (INT 90), ovariectomized - 90 days (OVX 90) and ovariectomized + resveratrol - 90 days (OVX + RES 90). The number of days in each group represented the duration of the experimental period. The animals in the intact groups received no intervention; the ovariectomized groups were ovariectomized and treated with a 0.9% saline solution (0.1ml/100g of body weight per day) by gavage until the end of the experimental period. Those in the ovariectomized + resveratrol group were ovariectomized and treated daily with a solution of 10mg/kg of body weight of resveratrol per day (solubilized in ethanol and diluted with distilled water, with the final concentration of ethanol at 5%), also by gavage for 60 or 90 days. At the end of the experimental period, the rats were anaesthetized with isoflurane and euthanized by decapitation. Blood and aorta artery were collected for experimental analysis.

#### Blood pressure

Systolic blood pressure (SBP) was measured by tail-cuff plethysmography (model Power Lab 8/35, AD Instruments, Pty Ltda, Colorado Springs, CO) in non-anesthetized animals, as described elsewhere by Rodrigues et al.,^12^ two days before the animals were killed by decapitation at the end of each experimental period. The average of four consecutive measurements was taken as the mean systolic blood pressure of each animal.

#### Vascular reactivity studies

The thoracic aortas were isolated, cleaned of adherent connective tissues, and placed in Krebs solution, as described elsewhere.^13^ The aortas were carefully dissected and mounted as ring preparations (≅4 mm in length) and placed in bath chambers (5 mL) containing Krebs solution at 37 °C (NaCl 130mM, KCl 47 mM, KH_2_PO_4_ 1.2 mM, CaCl 1.6; MgSO_4_ 1.2mM; NaHCO_3_ 14.9 mM; glucose 5.5 mM) continuously bubbled with 95% O_2_ and 5% CO_2_, pH 7.4, in a Mulvany-Halpern isometric myograph (model 610 DMT-USA, Marietta, GA) and recorded by a PowerLab8/SP data acquisition system (AD Instruments Pty Ltd., Colorado Springs, CO). The aortic rings were submitted to a tension of 1.5 g, which was readjusted every 15 min for a 60-min equilibration period before addition of the given drug. Experiments were conducted in aortic rings with intact endothelium and also in endothelium-denuded aortic rings. Endothelial integrity was assessed by the degree of relaxation induced by 1*µ*mol/l acetylcholine (Ach) in the presence of contractile tonus induced by phenylephrine (0.1*µ*M). The ring was considered as with intact endothelium if relaxation with acetylcholine was higher than 80%. In endothelium-denuded aortas, the relaxation to Ach was lower than 5%. After the endothelial integrity test, aortic rings were pre-contracted with phenylephrine (0.1*µ*M). When the plateau was reached, concentration-effect curves to acetylcholine (0.1nM to 0.1mM) in intact endothelium aortic rings or to sodium nitroprusside (SNP) in endothelium-denuded aortic rings were constructed. The potency (pD_2_) and the maximal relaxant effect (ME) were measured.

### Serum Nitrite and Nitrate (NOx)

Serum nitric oxide levels were obtained by measuring the serum concentrations of its stable end-products nitrite (NO_2_^-^) and nitrate (NO_3_^-^), collectively known as NO_x_. The NO/ozone chemiluminescence method was performed using the NO Analizer 280i (Sievers, Boulder, CO, USA). The NO_x_ concentration was corrected by the factor obtained by the quotient of the measured NO_x_ and expected concentrations of sodium nitrate (5, 10, 25, 50, and 100 *µ*M), yielding a standard curve.^14^

### Statistical analysis

Normality of distribution of the variables studied (all quantitative and continuous) was verified by the Kolmogorov-Smirnov test. Differences in means among the groups in each experimental period were compared by one-way analysis of variance (ANOVA). When significance was indicated, a Newman-Keuls post hoc analysis was used with statistical significance set at p<0.05 (Software Statistica 7.0, StatSoft. Inc, Tulsa, USA).

### Drugs and chemicals

Acetylcholine, phenylephrine and sodium nitroprusside, were purchased from Sigma-Aldrich (St.Louis, MO, USA). Resveratrol was purchased from Cayman Chemical (Ann Arbor, MI, USA).

## Results

In Table 1 we can observe that 60 days of ovariectomy did not change the endothelium-dependent and independent vascular relaxation of aortic rings, and resveratrol supplementation had no effect in the OVX group. The maximal relaxant effect (ME) did not change in aortic rings with or without endothelium for all groups. Also, a decrease in the potency of acetylcholine in inducing relaxation (pD2 OVX 90: 6.99 ± 0.10) was observed after 90 days of ovariectomy when compared to intact animals (pD2 INT 90: 7.51 ± 0.07, p<0.05). Ninety days of resveratrol supplementation was able to increase the pD2 to acetylcholine (pD2: OVX+RES: 7.50 ± 0.15, p <0.05) and also bring it to values similar to those of the intact groups, normalizing the endothelial function. In denuded aortic rings, no change was observed in the endothelium-independent relaxant effect in pD2 values in all groups. ME did not change after 90 days of ovariectomy or resveratrol supplementation in endothelium-dependent and independent relaxation induced by acetylcholine or sodium nitroprusside, respectively.

**Table 1:** Values of power (pD2) and maximal relaxant effect (ME) to relaxation induced by acetylcholine and sodium nitroprusside, in aortic rings with (E+) or without (E-) its endothelium from intact (INT), ovariectomized (OVX) and ovariectomized + resveratrol (OVX + RES) groups in both experimental periods. Values are expressed as Mean±SD. Comparisons were made using One-way ANOVA followed by the Newman-Keuls post- hoc test. *p<0.05 compared to INT 60 group; +p<0.05 compared to INT 90 group; # p< 0.05 compared to OVX 90 group

Relaxation induced by acetyilcholine (E+) and sodium nitroprusside (E-)			
**60 DAYS**	**INT 60**	**OVX 60**	**OVX+ RES 60**
pD2 E+	7.69 ± 0.15	7.43 ± 0.18	7.63 ± 0.16
ME E+	94.28 ± 4.80	84.66 ± 4.93	89.00 ± 4.43
pD2 E-	8.55 ± 0.09	8.51 ± 0.11	8.56 ± 0.09
ME E-	105.40 ± 2.12	103.30 ± 2.17	105.50 ± 2.71
**90 DAYS**	**INT 90**	**OVX 90**	**OVX+ RES 90**
pD2 E+	7.51 ± 0.07	7.00 ± 0.10^+^	7.50 ± 0.15^#^
ME E+	86.18 ± 4.32	85.50 ± 2.45	81.67 ± 3.61
pD2 E-	8.45 ± 0.02	8.45 ± 0.02	8.43 ± 0.01
ME E-	105.70 ± 2.62	105.20 ± 1.76	102.20 ± 4.21

**Figure 1:**
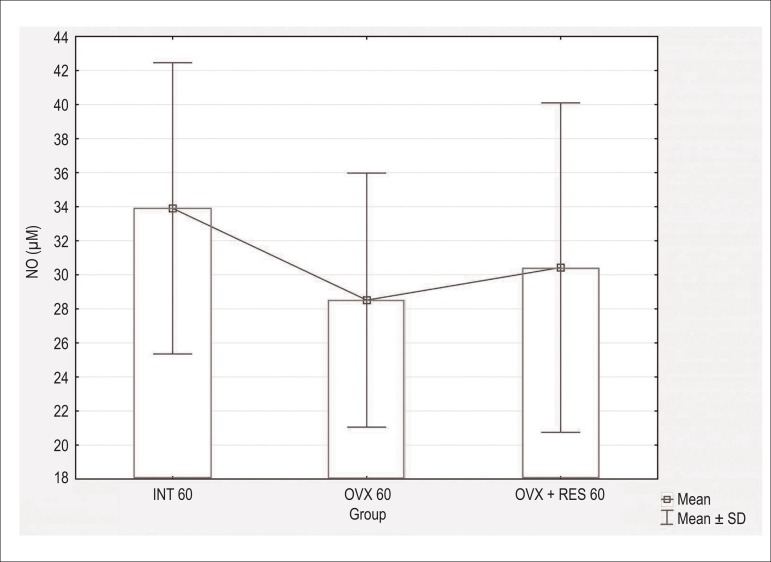
Serum nitric oxide concentration in µM in intact - 60 days (INT 60), ovariectomized - 60 days (OVX 60) and ovariectomized + resveratrol - 60 days (OVX + RES 60) groups. Values expressed as Mean±SD. Comparisons were made using One-way ANOVA followed by the Newman-Keuls post- hoc test. No differences were observed between the groups.

**Figure 2:**
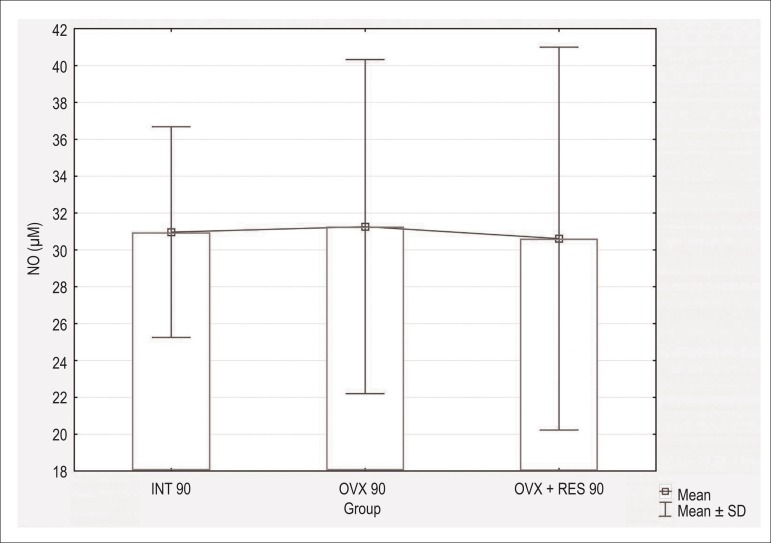
Serum nitric oxide concentration in µM in intact - 90 days (INT 90), ovariectomized - 90 days (OVX 90) and ovariectomized + resveratrol - 90 days (OVX + RES 90) groups. Values are expressed as Mean±SD. Comparisons were made using One-way ANOVA followed by the Newman-Keuls post- hoc test. No differences were observed between the groups.

In Table 2 we can observe that ovariectomy induced an increase in systolic blood pressure (SBP) 60 and 90 days after surgery. The treatment with resveratrol for 60 days did not prevent the increase in blood pressure. However, 90 days of treatment with resveratrol prevented it, and normalized blood pressure. Nevertheless, no difference could be observed in serum NO concentration (Figures 1 and 2) in both treatment periods (60 and 90 days).

**Table 2:** Systolic blood pressure (SBP) and serum Nitric Oxide concentration (NO) in intact (INT), ovariectomized (OVX) and ovariectomized + resveratrol (OVX + RES) groups of both experimental periods. Values expressed as Mean±SD. Comparisons were made using One-way ANOVA followed by the Newman-Keuls post- hoc test. *p<0.05 compared to INT 60 group; +p<0.05 compared to INT 90 group; # p< 0.05 compared to OVX 90 group

**60 DAYS**	**INT 60**	**OVX 60**	**OVX+ RES 60**
SBP (mmHg)	120.39 ± 4.58	138.16 ± 5.42*	135.18 ± 5.42*
NO (uM)	33.91 ± 8.55	28.51 ± 7.47	30.42 ± 9.68
**90 DAYS**	**INT 90**	**OVX 90**	**OVX+ RES 90**
SBP (mmHg)	123.92 ± 4.98	145.21 ± 9.79+	123.33 ± 3.66#
NO (uM)	30.96 ± 5.17	31.26 ± 9.06	30.61 ± 10.38

## Discussion

The main finding of this study was that the treatment with resveratrol for 90 days prevented the changes in blood pressure and endothelial function induced by estrogen deficiency. In this trial period, we have verified that ovariectomy was effective to induce endothelial dysfunction and elevation of blood pressure. The 60-day estrogen deficiency was not enough to induce changes in endothelial function in aortic ring of rats; however, this period was enough to increase the blood pressure value and resveratrol treatment did not modify endothelial function and blood pressure. 

The increase in blood pressure due to ovariectomy and its subsequent reduction in the group treated with resveratrol in the 90-day experimental protocol also was observed previously by Patki et al,^15^ who treated Wistar ovariectomized rats with frozen grape powder (in which one of the components is resveratrol). Still, the authors suggest that the effect of ovariectomy on blood pressure is induced by elevation in oxidative stress triggered by estrogen deficit and the effect of frozen grape powder may be related to its strong antioxidant effect,^15^ a feature also verified with resveratrol.^16^

The decrease in endothelium-dependent relaxation in aortic rings and consequent increase with the treatment with resveratrol in the 90-day experimental protocol agrees with the results presented by Mizutani and colleagues^10^ in stroke-prone spontaneously hypertensive ovariectomized rats, supplemented dietetically with 5mg/kg of body weight of resveratrol. However, these authors indicate that the effect of the substance on the endothelium is through the increased bioavailability of NO, as reported by other studies^17,18^, a fact not confirmed by our study.

An interesting result was that only prolonged resveratrol treatment (90 days) was able to improve the endothelial function and normalize blood pressure. Sixty days after surgery, no endothelium dysfunction was verified, and no improvement was induced by resveratrol. Thus, our result suggests that the improvement in the endothelial function induced by resveratrol normalizes the blood pressure in OVX rats by a NO independent mechanism. 

Vanhoute et al^4^ point out that in addition to NO there are other endothelium factors which can induce vasodilation, including the *endothelium*-derived hyperpolarizing factor (*EDHF*). Furthermore, Dolinsky et al^11^ suggested that the effect of resveratrol on blood pressure can be different in accordance to the experimental model used, and these differences could result from the distinct mechanisms of hypertension developing. Considering that there are few studies that have evaluated the effect of estrogen deficiency on blood pressure and endothelial function in young/adult animal models, the results of this study represent an important contribution of resveratrol as a preventive treatment for postmenopausal cardiovascular effects.

## Conclusion

Our results suggest that ninety days of treatment with resveratrol (10 mg/kg body weight per day) is able to normalize the endothelial function and blood pressure of ovariectomized rats via a NO-independent mechanism.

## References

[1] Belin de Chantemele EJ, Stepp DW (2012). Influence of obesity and metabolic dysfunction on the endothelial
control in the coronary circulation. J Mol Cell Cardiol.

[2] Cerqueira NF, Yoshida WB (2002). Óxido nítrico: revisão. Acta Cir Bras.

[3] Usselman CW, Stachenfeld NS, Bender JR (2016). The molecular actions of estrogen in the regulation of vascular
health. Exp Physiol.

[4] Vanhoutte PM, Shimokawa H, Feletou M, Tang EH (2017). Endothelial dysfunction and vascular disease - a 30th anniversary
update. Acta Physiol (Oxf).

[5] Macedo JM, Macedo CR, Elkis H, De Oliveira IR (1998). Meta-analysis about efficacy of anti-resorptive drugs in
post-menopausal osteoporosis. J Clin Pharm Ther.

[6] Ghazal S, Pal L (2013). Perspective on hormone therapy 10 years after the
WHI. Maturitas.

[7] Gehm BD, McAndrews JM, Chien PY, Jameson JL (1997). Resveratrol, a polyphenolic compound found in grapes and wine, is
an agonist for the estrogen receptor. Proc Natl Acad Sci U S A.

[8] Bhat KP, Kosmeder JW 2nd, Pezzuto JM (2001). Biological effects of resveratrol. Antioxid Redox Signal.

[9] Su JL, Yang CY, Zhao M, Kuo ML, Yen ML (2007). Forkhead proteins are critical for bone morphogenetic protein-2
regulation and anti-tumor activity of resveratrol. J Biol Chem.

[10] Mizutani K, Ikeda K, Kawai Y, Yamori Y (2000). Resveratrol attenuates ovariectomy-induced hypertension and bone
loss in stroke-prone spontaneously hypertensive rats. J Nutr Sci Vitaminol (Tokyo).

[11] Dolinsky VW, Chakrabarti S, Pereira TJ, Oka T, Levasseur J, Beker D (2013). Resveratrol prevents hypertension and cardiac hypertrophy in
hypertensive rats and mice. Biochim Biophys Acta.

[12] Rodrigues GJ, Pereira AC, Vercesi JA, Lima RG, Silva RS, Bendhack LM (2012). Long-lasting hypotensive effect in renal hypertensive rats
induced by nitric oxide released from a ruthenium complex. J Cardiovasc Pharmacol.

[13] Oishi JC, Buzinari TC, Pestana CR, De Moraes TF, Vatanabe IP, Wink DA Jr (2015). In vitro treatment with cis-[Ru(H-dcbpy-)2(Cl)(NO)] improves the
endothelial function in aortic rings with endothelial
dysfunction. J Pharm Pharm Sci.

[14] Pereira FH, Batalhão ME, Cárnio EC (2014). Correlation between body temperature, blood pressure and
plasmatic nitric oxide in septic patients. Rev Lat Am Enfermagem.

[15] Patki G, Allam FH, Atrooz F, Dao AT, Solanki N, Chugh G (2013). Grape powder intake prevents ovariectomy-induced anxiety-like
behavior, memory impairment and high blood pressure in female Wistar
rats. PLoS One.

[16] Frombaum M, Le Clanche S, Bonnefont-Rousselot D, Borderie D (2012). Antioxidant effects of resveratrol and other stilbene derivatives
on oxidative stress and *NO bioavailability: potential benefits to
cardiovascular diseases. Biochimie.

[17] Breen DM, Dolinsky VW, Zhang H, Ghanim H, Guo J, Mroziewicz M (2012). Resveratrol inhibits neointimal formation after arterial injury
through an endothelial nitric oxide synthase-dependent
mechanism. Atherosclerosis.

[18] Yamagata K, Tagami M, Yamori Y (2015). Dietary polyphenols regulate endothelial function and prevent
cardiovascular disease. Nutrition.

